# Cardiac Auscultation Lab Using a Heart Sounds Auscultation Simulation Manikin

**DOI:** 10.15766/mep_2374-8265.10839

**Published:** 2019-10-18

**Authors:** Antonia Quinn, Jennifer Kaminsky, Andrew Adler, Shirley Eisner, Robin Ovitsh

**Affiliations:** 1 Associate Clinical Professor, Department of Emergency Medicine, State University of New York Downstate Medical Center; 2 Associate Director of Clinical Competencies, State University of New York Downstate College of Medicine; 3 Fourth-Year Medical Student, State University of New York Downstate College of Medicine; 4 Professor, Department of Medicine, State University of New York Downstate Medical Center; 5 Associate Professor, Department of Cell Biology, State University of New York Downstate College of Medicine; 6 Associate Professor, Department of Pediatrics, State University of New York Downstate Medical Center; 7 Associate Dean for Clinical Competencies, State University of New York Downstate College of Medicine

**Keywords:** Cardiac Auscultation, Deliberate Practice, Simulation, High Fidelity, Murmurs, Heart Sounds, Clinical Skills, Curriculum Development, Case-Based Learning, Laboratory Education

## Abstract

**Introduction:**

Cardiac auscultation skills are essential to the development of a competent physician. We created a hypothesis-driven cardiac auscultation laboratory session utilizing a high-fidelity simulator to teach these skills to second-year medical students at our institution. This program was grounded in deliberate practice opportunities to aid in the acquisition of cardiac auscultation skills.

**Methods:**

This session aimed to help students identify and discriminate between normal and pathologic heart sounds in the context of a clinical vignette. Faculty facilitators guided students through unknown patient cases and utilized the auscultation manikin to simulate corresponding heart sounds. Time was also allotted for students to auscultate the manikins and practice their cardiac physical examination skills.

**Results:**

This program has been in place at our institution since 2016 and has been well received by students and facilitators. Since its initial introduction in 2016, 183 second-year medical students have completed the cardiac auscultation lab session each year, for a total of 549 students. Evaluations of the session have improved as faculty have become more familiar with the mechanics of operating the auscultation manikin.

**Discussion:**

The cardiac exam and heart sounds lab can be adapted to any simulator model that is capable of producing heart sounds and can be done in a large- or small-group format. Enough time should be allotted to adequately work through all components of the laboratory. Student and faculty feedback has helped us further refine the session since its initial introduction to the curriculum.

## Educational Objectives

By the end of this module, learners will be able to:

1. Describe S1 and S2 with respect to intensity, location, and splitting.
2. Identify and interpret physiologic and pathologic S1 and S2 including splitting in the context of a patient scenario and/or disease.
3. Identify S3 and S4 gallops and interpret S3 and S4 gallops in the context of a patient scenario and/or disease.
4. Describe location, timing, and intensity of common systolic and diastolic heart murmurs.
5. Identify and interpret different systolic and diastolic murmurs in the context of a patient scenario and/or disease.
6. Demonstrate and distinguish auscultatory use of the diaphragm and bell of the stethoscope.

## Introduction

Although cardiac auscultation is a core clinical skill, the ability to recognize and interpret heart sounds is in decline across a range of medical specialties.^1^ Studies have shown that the use of simulation-based technology coupled with deliberate practice opportunities leads to superior acquisition of clinical skills^2^ and improves competency. Deliberate practice emphasizes the purposeful, focused learning of a particular skill; rather than having learners mechanically run through a task, they are provided with guided practice opportunities, feedback, and time to solve relevant problems.^3^ Our program utilizes this approach in teaching cardiac auscultation to students at our institution. Faculty guide and coach students through the material and provide constructive feedback with the specific goal of improving auscultatory discrimination and reasoning skills. Learners are able to apply feedback throughout each component of our session and in subsequent review to further enhance their understanding of heart sounds.

Simulation can help to improve the transfer of cardiac auscultation skills from the classroom to the clinical setting.^4^ As digital natives, current students may also feel more comfortable with simulation technology; students are able to interact and engage with their immediate environment via simulation opportunities, further aligning with how they think and process information. Using the auscultation manikin, students learn heart sounds through hands-on practice in an environment most similar to actual patient care. Utilization of a torso in practice has been shown to increase knowledge retention and acquisition of heart sounds through deliberate and repetitive practice.^5^

To improve our cardiac auscultation training, we replaced a commercially available web-based cardiac auscultation program with the SAM II cardiac auscultation manikin (Cardionics, Webster, Texas). Prior to utilizing the auscultation manikin, both student and faculty reviews of the heart sounds lab were fair. Many students and facilitators felt that using a free commercial online program was cumbersome to navigate and that the sound quality was often poor. Pop-up advertisements were distracting as well.

Unlike the web-based commercial product, the auscultation manikin produces normal and pathologic heart sounds in an authentic manner and provides students an opportunity to practice their cardiac auscultation skills. Because the manikin is a half torso, students are able to auscultate at the anatomically correct position for each heart sound, as the manikin accurately aligns with human surface anatomy. This allows for the integration of a physical model into the learning experience. Finally, the auscultation manikin can produce cardiac and respiratory sounds in combination, allowing the learner to hear an authentic reproduction of cardiopulmonary physiology and pathophysiology.

Through our program, students work through clinical vignettes in conjunction with simulated heart sounds and are prompted to anticipate auscultatory findings for each case, reflecting a hypothesis-driven approach. Traditionally, the physical examination is taught in a head-to-toe format, whereas the hypothesis-driven physical examination (HDPE) emphasizes the importance of approaching a physical examination with a differential diagnosis in mind. Through a hypothesis-driven approach, students are taught to utilize physical examination maneuvers to determine the likelihood of pathology. As opposed to teaching the physical examination by rote maneuvers, the HDPE approach has been shown to increase learner retention and understanding of the significance of physical findings.^6^

Our search in *MedEdPORTAL* yielded only two prior curricula on the teaching of cardiac auscultation to medical students,^7,8^ neither of which focus on the use of high-fidelity simulation to teach heart sounds. Furthermore, one of the curricula is out of print.^7^ This report describes our revised cardiac auscultation program design and preliminary results for student and faculty evaluation and general acceptance of this new innovation to the curriculum.

## Methods

### Target Audience

The target audience was second-year preclinical medical students. Although this session was created for medical students, it could also be used by other health professions students (e.g., physician assistant, nursing) or residents, adjusting the level of difficulty as appropriate for the learner.

### Prerequisites

• Introduction and understanding of basic normal heart sounds.
• Understanding of surface anatomy and location of underlying valves.

### Preparation

Prior to the session, we programmed the cardiac simulator to produce the heart sounds in the sequence that we chose to utilize for the session (Appendix A). We began with physiologic heart sounds to provide a foundation and progressed to pathologic heart sounds and murmurs. Each institution should program the simulator according to the manufacturer's instructions.

Faculty facilitators were physicians from various medical specialties, including internal medicine, emergency medicine, and pediatrics. However, this session could be facilitated by any experienced clinician (e.g., resident physician, physician assistant, or nurse practitioner) with a background in cardiac auscultation skills. The recruitment of facilitators will be institution specific depending on how physical examination skills are taught. We used resident and attending physicians to teach this session at our institution, with one faculty member per 12 students. One near-peer educator (NPE), a senior medical student who worked alongside faculty to aid in facilitation of physical examination labs, was also assigned to each group of students. Although a tremendous asset, an NPE is not required for success of the session. There was one auscultation manikin per 12 students.

Facilitators received approximately 1 hour of faculty development introducing them to the mechanics of the simulator and how to progress through the sounds. Faculty reviewed each vignette and were provided with a list of probing questions with answers to enhance their facilitation of the lab (Appendix B). There were no formal faculty development materials aside from the facilitator manual. The majority of the faculty development involved practice auscultating the manikin, using the laptop computer, and presenting the probing questions.

In preparation for the lab, students had the opportunity to attend an interactive lecture by one of our faculty cardiologists utilizing the heart sounds simulator; the cardiologist commented on the manikin's marked fidelity to true human heart sounds. Lectures were not mandatory at our institution, so the onus of prereading and preparation for the lab was on the students. *Bates’ Guide to Physical Examination and History Taking*^9^ was the required physical examination textbook at our institution; however, any physical examination textbook (e.g., *DeGowin's Diagnostic Examination*^10^) would be acceptable for prereading for the session.

### Session Logistics

This lab utilized the SAM II cardiac auscultation manikin, but it could easily be adapted to any auscultation simulator, such as Harvey (Laerdal Medical, Wappingers Falls, New York) and Ventriloscope (Lecat's Ventriloscope, Tallmadge, Ohio). The SAM II model was a portable torso that sat upright on a base and plugged into a wall electrical outlet. The model contained internal speakers; it attached to a laptop via USB cables and could be projected onto a video screen.

This session was held during our regularly scheduled physical diagnosis laboratory. If room and time constraints are an issue, this module could be adapted to a large-group session. However, the students would not have the opportunity to auscultate the manikin directly. Conversely, the cases could be used as a case-based direct auscultation simulation session or objective structured clinical examination (OSCE). The time allotted for the lab was 2 hours, which also included cardiac physical examination practice. This publication provides the materials for the heart sounds lab portion utilizing the auscultation manikin. Even though our session was held during the cardiovascular block of our curriculum, it could be a stand-alone session whenever the calendar permits. Unlike other physical examination maneuvers, cardiac auscultation does not need to be taught in an integrated, case-based fashion but rather can be learned as an isolated skill.^5^

During the lab, students were asked to identify and interpret various simulated heart sounds. We interchanged the pathologic sounds with normal sounds to enhance auditory discrimination and improve auscultation skills. Students were provided with the vignettes and probing questions (with the answers removed) to allow them to anticipate auscultatory findings during the session (Appendix C).

#### One hour:

The lab employed a case-based, hypothesis-driven approach to cardiac auscultation. Students were presented with eight unknown clinical scenarios. The facilitators were provided with both the scenarios and their correct interpretations. Prior to playing the associated heart sounds, the facilitator asked the students which heart sounds they would anticipate. The facilitator then played the sounds on the simulator and asked a series of related probing questions. Examples included location for best auscultation of specific murmurs, diagnoses associated with particular heart sounds, and pathophysiology of murmurs. The SAM II program also depicted the surface location and the associated phonocardiogram on a projected video screen using a VGA connection to a laptop.

#### Forty minutes:

The cardiac auscultation manikin had internal speakers, which allowed the learners to directly auscultate the simulator using their own stethoscopes. Two pairs of students utilized the manikin for 10 minutes to evaluate a simulated patient vignette, this time auscultating the manikin directly and analyzing the heart sounds. The remaining students practiced the physical examination on each other during that time while waiting to use the simulator. The timing and organization could be adjusted to the needs and time constraints of the individual institution using this module.

Students were provided with the answers to the probing questions 1 week after the session (Appendix D). The questions were written in a format that allowed the students to quiz themselves for spaced repetition to optimize long-term retention and recall of the material.

This laboratory was evaluated by a course/faculty assessment team (a core group of 20 students tasked with evaluating lecture, laboratory, and small-group sessions, as well as the instructors) at the end of the week immediately following the session. Feedback on the session was then presented to clinical skills leadership. The lab was also evaluated by all students at the end of the cardiovascular curricular block. Students were evaluated via an end-of-unit OSCE, in which they listened to the cardiac simulator and answered questions identifying and describing their auscultatory findings and the associated clinical significance. There were also four multiple-choice questions pertaining to the educational objectives on the end-of-unit exam.

## Results

Since the session's initial introduction in 2016, 183 second-year medical students have completed it each year, for a total of 549 students. The session was conducted over the course of 2 days (with about 92 students each day) and further divided into two separate 2-hour blocks with 46 students in each block. We utilized four rooms for the session (six pairs of students in each room). Each of the 183 students was offered the opportunity to submit evaluation data at the end of the week and ranked the session on a 5-point Likert scale (Appendix E). Students also had the opportunity to enter additional comments on the bottom of their feedback form. This survey was a general curricular survey for all didactic sessions held during the week and was not specific to the heart sounds lab.

After introduction of the auscultation manikin, the reviews of the lab improved appreciably. When the session was first introduced, 35% of the students rated it as “very effective” and 26% as “extremely effective.” The third iteration of the program was held in 2018; student evaluations have continued to improve since the initial implementation. In 2018, 50% of the students rated the session as “very effective” and 35% as “extremely effective.”

Themes gleaned from specific comments about the program included that the auscultation manikin is an excellent modality for learning abnormal sounds and recognizing clinical presentations. Students and faculty commented on the ease of use of the manual and opportunity to review material. Constructive comments concerned variability of faculty experience and duration of the session. Specific comments about the program included the following:

• Positive comments:
  ○ “Excellent session. I learned a lot about abnormal heart sounds.”
  ○ “I really enjoyed the heart sounds lab and thought it was extremely useful in helping us start to recognize clinical presentations.”
  ○ “Good way to help with getting us to hear the heart sounds; without this, I had no idea what to listen to besides S1 and S2.”
  ○ “The manual was nicely written.”
• Constructive comments regarding session duration:
  ○ “Physical exam labs and clinical skills sessions (with SAM II) were excellent. I wish they had been longer because they were so well-organized, we were just a bit rushed.”
  ○ “I loved the heart sounds lab. I only wish it were longer or split up into two sessions so we had time to practice in between and revisit it.”
  ○ “The heart sounds/cardiac exam lab was variable and depending on who your facilitator was, the session could be very helpful or less so.”

Course/faculty team members also provided feedback on the session duration, noting that it would be helpful for students to have more hands-on time with the manikin.

## Discussion

We revised our cardiac examination lab by implementing a high-fidelity manikin to teach cardiac auscultation. We found that overall, the sessions were well received by faculty and students. Since the introduction of the session 3 years ago, no changes have been implemented to the teaching methods because of very favorable reviews. The most significant change has been increasing the time of the session from 1.5 to 2 hours. Some of the more challenging and less classic cases have been made optional, such as hypertrophic obstructive cardiomyopathy, in the interest of time. We recommend extending the duration of the session even further where curriculum time permits.

Adequate time should be dedicated to the group cases and individual auscultation practice with the manikins. Although this lab could be separated into two distinct sessions (e.g., the physical examination and group cases in one time slot, individual auscultation practice in a second time slot), further faculty recruitment, administrative support, classroom space, and so forth would be required. Additionally, because SAM II can be expensive, an institution may be able to purchase only one simulator. This session can be adapted to other high-fidelity models, such as Harvey or Ventriloscope, or can be done in a large-group setting, which would only require one manikin. However, this would prevent students from gaining individual hands-on practice time.

We had robust faculty-student ratios for our sessions. Therefore, we believe that the students’ concern that there was not enough time for the session was an issue of time allotted for hands-on practice rather than lack of supervision and feedback.

Reviews of the session by facilitators have improved over the past few years; a core group of faculty has been involved with the session since it was first introduced, likely increasing their familiarity with the technology. The improvement in student evaluations mirrors the comfort of the facilitators. Faculty new to the session this year expressed feeling less comfortable with the simulators than did our more experienced group of facilitators despite training regarding the mechanics of operating the manikin.

### Limitations

One of the limitations of this laboratory is that students were not held accountable to complete prereading beforehand; this could hinder opportunities for deliberate practice and skill improvement.

Our session is also limited by a lack of evaluation on our objectives. Even though we have four multiple-choice questions on our end-of-unit summative exam related to our learning objectives and an OSCE, there is not enough data to assess at this point whether the knowledge is retained in actual clinical practice.

Evaluations of the session were based primarily on student and faculty perception and are level one of Kirkpatrick's learning pyramid.^11^ Although student evaluations have improved considerably since the initial introduction of the SAM II lab, many students indicated that they felt they did not have enough time to directly auscultate and utilize the manikins during the laboratory session despite the increase in the duration of the session.

### Future Opportunities

Opportunities for future improvement could entail lengthening the duration of the session to 3 hours to address students’ desire for more individual auscultation time with the manikin. The time allotted for this session in our curriculum is 2 hours; however, other institutions could make alterations to this as scheduling permits. We also suggest adding in additional time for review (through after-hours review sessions or revisiting the session later in the academic year, if resources are available) to encourage spaced repetition and reinforcement of the material; this would allow students to practice their cardiac auscultatory skills before beginning third-year clinical coursework. Furthermore, as there is no presession readiness assessment at our institution for this session, other institutions could implement a pretest or other required presession assignment to ensure a baseline level of knowledge among learners.

## Appendices

A. Heart Sounds - Programming List.docx
B. Heart Sounds Lab - Facilitator Manual.docx
C. Heart Sounds Lab - Student Manual.docx
D. Post-Heart Sounds Lab Discussion.docx
E. Session Feedback Form.docx All appendices are peer reviewed as integral parts of the Original Publication.
