# Supplementary material for: Cardiac Auscultation Lab Using a Heart Sounds Auscultation Simulation Manikin
Source: MedEdPORTAL. 2019 Oct 18;15:10839. doi: 10.15766/mep_2374-8265.10839 (PMC6974355; doi:10.15766/mep_2374-8265.10839)
Supplement: Supplementary file 1 — A. Heart Sounds - Programming List.docx B. Heart Sounds Lab - Facilitator Manual.docx C. Heart Sounds Lab - Student Manual.docx D. Post-Heart Sounds Lab Discussion.docx E. Session Feedback Form.docx [file mep-15-10839-s001.zip › A. Heart Sounds - Programming List .docx]

Please refer to the following list when programming SAM II manikins. Heart sounds are listed in order of cases in the ‘Cardiac Exam and Heart Sounds Lab’ manual.

Group cases:

1. Normal Heart Sounds-S1/S2 unsplit
2. Normal Heart Sounds-S1/S2 physiologic splitting
3. S3 heart sound
4. S4 heart sound
5. Innocent (Functional) Murmur
6. Aortic Stenosis
7. Aortic Regurgitation
8. Hypertrophic Cardiomyopathy (HOCM)

Individual auscultation practice with SAM II:

1. Mitral Regurgitation
2. Mitral Stenosis
