# Supplementary material for: Cardiac Auscultation Lab Using a Heart Sounds Auscultation Simulation Manikin
Source: MedEdPORTAL. 2019 Oct 18;15:10839. doi: 10.15766/mep_2374-8265.10839 (PMC6974355; doi:10.15766/mep_2374-8265.10839)
Supplement: Supplementary file 1 — A. Heart Sounds - Programming List.docx B. Heart Sounds Lab - Facilitator Manual.docx C. Heart Sounds Lab - Student Manual.docx D. Post-Heart Sounds Lab Discussion.docx E. Session Feedback Form.docx [file mep-15-10839-s001.zip › B. Heart Sounds Lab - Facilitator Manual.docx]

**Facilitator Manual: Cardiac Exam and Heart Sounds Lab**

**Body Systems II: Cardiovascular, Respiratory, and Renal Systems**

**Session Title: Clinical Skills Lab: Cardiac Exam and Heart Sounds**

**Description**

- Coaching and practicing the skill of performing the cardiac exam
- The session will begin with a lab and discussion on heart sounds.

**Readings**

Bates’ Guide to Physical Examination and History Taking, 11th edition

pp  333-344 (Surface anatomy, cardiac chambers/valves, cardiac cycle, heart sounds, S3, S4, conduction system)

pp  360-366 (Techniques of Examination)

pp  367-368 (Carotid pulse, pulsus alternans, paradoxical pulse, thrills and bruits)

pp  369-385 (Heart Examination)

pp  500-504 (Peripheral pulses of arms and legs)

Heart Sounds:

<http://www.blaufuss.org> (optional)

Use this valuable web page to explore the different heart sounds and murmurs to understand the relevant anatomy and pathophysiology.

**Learning Objectives:**

By the end of this module, the learner will be able to:

1. Describe S1 and S2 with respect to intensity, location and splitting.
2. Identify and interpret physiological and pathological S1 and S2 including splitting in the context of a patient scenario and/or disease.
3. Identify S3 and S4 gallops and interpret S3 and S4 gallops in the context of a patient scenario and/or disease.
4. Describe location, timing and intensity of common systolic and diastolic heart murmurs.
5. Identify and interpret different systolic and diastolic murmurs in the context of a patient scenario and/or disease.
6. Demonstrate and distinguish auscultatory use of the diaphragm and bell of the stethoscope.

**Equipment**: Stethoscope

**Pre/Post-session assignments:**

No pre-session assignments

For further practice after the session and review of murmurs not covered in the lab, go to: [www.blaufuss.org](http://www.blaufuss.org) (optional)

**PHYSICAL EXAM SESSION FORMAT:**

Your students will have 120 minutes to learn and practice the technique of performing a cardiac exam.

**0-10 minutes: Sign-in, brief orientation**

- Brief introduction of group members and facilitator
- Review of basic cardiac exam

**10-70 minutes: Heart Sounds Lab utilizing SAM II Cardiac Auscultation Manikin**

**70-110 minutes: Practice cardiac exam using checklist and get feedback on skill; Practice cases utilizing SAM II Cardiac Auscultation Manikin**

- Students should pair up and find an examination table on which to examine their partner.
- Students should practice their interpersonal skills by examining their partner in the role of a physician.
- Students will utilize the auscultation manikin as a team of 4 (2 student pairs) to further analyze two additional cases.

**110-120 minutes: Debrief**

- Review the strengths and difficulties encountered by the students during the exam.
- Wrap up and final thoughts

Rate?

(Fast/Normal/

Slow)

Rhythm?

(Regular, Irregular)

Murmur?

(Yes or No)

+ Murmur

Location?

Systolic/Diastolic?

Shape?

Extra Sounds?

(Systole/Diastole)

# Cardiac Auscultation Algorithm

The SAM II Cardiac Auscultation Manikin is the basis for this heart sounds lab. Below are the steps to locating the audio for a variety of normal and abnormal heart sounds. Each section is introduced with a small case and is followed by discussion questions that should be answered while examining each of the heart sounds. In order to improve clinical reasoning, have the students predict which heart sounds are expected for each clinical scenario prior to listening to the sounds.

# Algorithm for Murmur Interpretation

Accentuated by Maneuver?

The heart sounds will be uploaded to the heart sounds auscultation simulator (SAM II) prior to the session. As you progress through the cases, use the forward arrow to advance to the next heart sound. It is recommended to press “pause lecture” once you click on a sound, otherwise the program will advance to the next sound after two minutes. To return to a previous sound, simply press the back arrow. To play the sound through the external speakers, place the arrow on the diagram of the thorax on the screen. Find the sound number on the bottom left of the screen.

To have students listen to the sounds through the manikin’s internal speakers, highlight the stethoscope on the screen. Please refer to the image below.





SAM II phonocardiogram projected on video screen

Image created by the Cardionics^®^ SAM system, reproduced with permission from Cardionics^®^ USA

**Case #1**

**Normal Heart Sounds-S1/S2 unsplit**

**(Sound #1 and 2)**

Case: Ms. Jennifer Alvarez who you had seen last year for her pre-exercise clearance is back for her routine yearly examination. You listen to her heart and hear the following:

Ask the students before playing the sound: “Based on the case what heart sounds do you expect to hear?” Normal S1/S2.

(Play Normal Sinus @ 75 bpm, Sound #1) What sound are you hearing? Normal Sinus at 75 bpm

Discussion Questions as you are examining the normal heart sounds

1. How can you tell the first heart sound from the second? At normal heart rates (below 100) systole is shorter than diastole so the first heart sound occurs after a longer pause. The first heart sound occurs right before the carotid pulse is felt.
2. Where is S1 best heard? The first heart sound is louder at the 5^th^ intercostal space (apex).
3. What does S1 signify? What is happening anatomically during S1? Closing of the mitral and tricuspid valves
4. What does S2 signify? What is happening anatomically during S2? Closing of the aortic and pulmonic valves
5. Where is the S2 best heard? At the 2^nd^ intercostal spaces (base)
6. (Play S1/S2, normal heart sounds at 110 bpm, Sound #2) What do you hear now? How is it different? (nml heart sound S1/S2 but at 110 bpm. Stress importance of listening to rate and rhythm).

**Case #2**

**Normal Heart Sounds-S1/S2 physiologic splitting**

**(Sound #3)**

As your auscultation skills improve, you listen to Ms. Alvarez’s heart and hear the following:

Discussion questions as you are examining the physiologic split of S2:

1. (Ask while playing audio Sound #3) What are you hearing? S1 with a Split S2
2. How does this heart sound differ from the previous case? The S2 is divided into 2 separate sounds that fuse into a single sound in deep expiration.
3. What position do you have the patient lying in to appreciate this sound the best? Supine
4. What part of the stethoscope would you use to hear this sound the best? Bell
5. Where do you place the stethoscope to best hear this sound? Over the pulmonic area (base)
6. What causes a split S2? Timing of the closure of the aortic and pulmonary valves (and therefore the components of the 2^nd^ heart sound) varies causing a split sound
7. Why does the S2 change with inspiration and expiration? Splitting occurs at maximum inspiration and is absent at expiration due to the timing of the closure of the aortic and pulmonary valves. Inspiration increases right sided filling thereby delaying pulmonic valve closure.

**Case #3**

**S3**

**(Sound #4)**

A 67-year-old man with a history of a myocardial infarction 6 months ago now presents with pedal edema, orthopnea, decreasing exercise tolerance with dyspnea on walking 2 blocks and you hear the following heart sounds:

Discussion questions as you are examining the S3 gallop:

1. Ask the students before playing the sound: Based on the case what heart sounds do you expect to hear? Pt has signs and symptoms of CHF. The students should consider an S3 gallop.
2. (Ask while playing audio Sound #4) What heart sounds are you hearing? S1/S2 and S3 gallop (Kentucky)
3. What is an S3? The third heart sound is caused by a sudden deceleration of blood flow into the left ventricle from the left atrium.
4. When in the cardiac cycle does an S3 occur? Early diastole
5. Where on the chest wall is an S3 best heard? Mitral area, apex of the heart
6. What part of the stethoscope do you use to best hear an S3 and why? The bell because it is a low-pitched sound
7. How is the patient positioned to best hear an S3? Left lateral decubitus position
8. What does the presence of an S3 indicate clinically in a pediatric patient or athlete? It may be normal.
9. What does the presence of an S3 indicate clinically in this patient? Congestive heart failure

**Case #4**

**S4**

**(Sound #5)**

A 72-year-old woman with long-standing hypertension presents for her routine examination. Her blood pressure is still elevated at 160/94. You hear the following heart sounds:

Discussion questions as you are examining S4 gallop:

1. Ask the students before playing the sound: Based on the case what heart sounds do you expect to hear? S4 gallop due to atrial contraction against a stiffened ventricle
2. (Ask while playing audio Sound #5) What heart sounds are you hearing? S1/S2 and S4 gallop (Tennessee)
3. What is an S4? A low-pitched diastolic gallop heard in pre-systole. It typically occurs about 90msec before S1. It is caused by a forceful atrial contraction against a stiffened ventricle that cannot expand any further.
4. When in the cardiac cycle does an S4 occur? Late diastole
5. Where on the chest wall is an S4 best heard? The 5^th^ intercostal space, midclavicular line just below the left nipple (apex)
6. What part of the stethoscope do you use to best hear an S4 and why? Bell because it is a low-pitched sound
7. How is the patient positioned to best hear an S4? Left lateral decubitus position
8. What does an S4 indicate clinically? The left ventricle is stiffened from hypertrophy of fibrosis.

**Case #5**

**Innocent (Functional) Murmur**

**(Sound #6)**

A 12-year-old boy with sickle cell disease is admitted with an acute painful crisis. He is anemic and his hemoglobin is 5.2gm/dl. While listening to his heart you hear the following:

Discussion questions for innocent murmur:

1. Ask the students before playing the sound: Based on the case what heart sounds do you expect to hear? Innocent high flow murmur due to severe anemia
2. (Ask while playing audio Sound #6) What are you hearing? An innocent murmur
3. When does an innocent murmur occur during the cardiac cycle? Functional murmurs are short early to mid-systolic murmurs
4. Where is an innocent murmur best heard? They are well localized to the left sternal border
5. What is the intensity or grading of functional murmurs? What does this mean? They are grade less than 2/6. This means they are low intensity and usually audible to inexperienced listeners. There is no thrill.
6. What maneuvers decrease the intensity of a functional murmur? Innocent murmurs decrease in intensity when the patient stands, sits up or strains during Valsalva maneuver.
7. What are some causes of functional murmurs? These murmurs are usually due to conditions outside of the heart- anemia, fever with increased blood flow.

**Case #6**

**Aortic Stenosis**

**(Sound #7 and #8)**

A 76-year-old woman with long-standing hypertension complains of substernal chest pain on walking 3 blocks that lasts for 5 minutes and then resolves when she stops walking. She also has dyspnea on exertion. While listening to his heart you hear the following:

Discussion questions for the murmur of aortic stenosis:

1. Ask the students before playing the sound: Based on the case what heart sounds do you expect to hear?
2. (Ask while playing audio of mild aortic stenosis Sound #7) What are you hearing in regards to heart sounds? S1 is normal. S2 is present. There is a systolic murmur between S1 and S2.
3. Describe the murmur. This is a late peaking (diamond shaped) systolic murmur.
4. Based on these findings (a diamond shaped midsystolic murmur) what is the diagnosis? Aortic stenosis
5. Where is this murmur usually heard and where does it tend to radiate? Over the right sternal border, 2^nd^ intercostal space. The murmur typically radiates to the carotids.
6. What would you find on palpating her PMI? A sustained apical impulse due to LVH.
7. What would you find on palpating her carotids? A small volume impulse and delayed (pulsus parvus et tardus).
8. (Ask while playing for marked AS Sound #8). Make sure to also place cursor on carotid radio button to hear the radiation of the murmur) The patient now returns several months later after passing out. How does this heart sounds differ from the previous one? Increased in intensity and radiates to carotids. As the disease progresses the intensity of S2 decreases. An absent S2 as in this case indicates severe disease.

**Case #7**

**Aortic Regurgitation**

**Sound #9**

A 46-year-old male recently emigrated from North Africa to the United States and wants to establish primary care. His past medical history is significant for rheumatic fever as a child. You listen to his heart and hear the following:

Discussion questions for the murmur of aortic regurgitation:

1. Ask the students before playing the sound: Based on the case what heart sounds do you expect to hear? Diastolic murmur of acute aortic regurgitation from endocarditis
2. What are you hearing regarding heart sounds? S1 is present with absent S2.
3. Describe the murmur. A blowing decrescendo diastolic murmur
4. Based on these findings (A high pitched decrescendo murmur) what is the diagnosis? Aortic regurgitation
5. What position should the patient be sitting in to best hear the murmur of AR? Seated leaning forward holding his breath after expiration
6. Where is this murmur usually heard and where does it tend to radiate? At Erb’s Point- the third intercostal space on the left sternal border where S2 is best auscultated. If loud, this murmur may radiate to the apex and along the left sternal border.

**Case #8**

**Hypertrophic Cardiomyopathy (HOCM) (use only if time permits)**

**Sound #10**

A 14-year-old boy is concerned because his 20-year-old cousin was recently told to stop playing College Varsity basketball. You listen to his heart and hear the following:

Discussion questions for the murmurs of hypertrophic cardiomyopathy:

1. Ask before playing audio: Based on the case what do you think the diagnosis may be?
2. (Ask while playing audio Sound #10) What are you hearing in regards to heart sounds? S1 is normal. S2 is normal. There may be an S4 due to the stiffness of the left ventricle
3. Describe the murmur. There is a harsh diamond shaped murmur that starts at the beginning of systole and ends well before the second heart sound. There are actually two murmurs, a systolic aortic murmur and a systolic mitral murmur. However, because they occur at the same time, they are heard as one murmur.
4. Based on these findings (a harsh shaped midsystolic murmur in the aortic area with a systolic murmur in the mitral area) what is the diagnosis? Hypertrophic Cardiomyopathy
5. Why are there 2 murmurs in this condition? The first systolic murmur results from the strong contraction of the stiff left ventricle causes the anterior leaflet to be sucked into the ventricle, blocking the flow into the aorta and causing an aortic murmur. At the same time, turbulent flow from the left ventricle to the left atrium causes a second systolic mitral murmur.
6. Where is this murmur usually heard and where does it tend to radiate? The rectangular shaped pansystolic murmur is best heard over the apex while the aortic midsystolic diamond shaped murmur is best heard and the left sternal border, 2^nd^ intercostal space.
7. What does handgrip, squatting and leg elevation do to this murmur? What about Valsalva strain? Why? The murmur would decrease in intensity with handgrip and squatting due to an increased left ventricular volume and arterial pressure. The intensity of the murmur decreases with increased afterload. Valsalva and moving from squatting to standing decreases blood return to the heart therefore decreasing ventricular volume. This increases the obstruction and increases the murmur’s intensity.

**SAM II auscultation manikin Case #1**

**Mitral Regurgitation**

**Sound #11 and #12**

A 35yo female with a history of mitral valve prolapse since her teenage years. She presents with worsening dyspnea on exertion, fatigue, and decreased exercise tolerance. You auscultate her heart and hear the following:

Discussion questions for the murmur of mitral regurgitation:

1. Ask before playing audio: Based on the case what do you think the diagnosis may be?
2. (Ask while playing audio of mild mitral regurgitation Sound #11) What are you hearing regarding heart sounds? S1 is normal. S2 is normal.
3. Describe the murmur. This is a holosystolic rectangular shaped murmur. It is located at the apex.
4. Based on these findings (a rectangular shaped holosystolic murmur) what is the diagnosis? Mitral regurgitation
5. What position should the patient be in to best hear the murmur of MR? Left lateral decubitus position
6. Where is this murmur usually heard and where does it tend to radiate? At the apex (midclavicular line 5^th^ intercostal space) and radiating to the axilla.
7. What would bilateral increased handgrip do to this murmur? Why? The murmur would increase in intensity by increasing arterial resistance causing more blood to flow back through the mitral valve.
8. (Play the audio for severe mitral regurgitation Sound #12) What do you hear now? How does the sound and radiation differ from the previous heart sound?

**SAM II auscultation manikin Case #2**

**Mitral Stenosis**

**Sound #13**

A 36yo female from Haiti presents to your office. She states she has a past medical history of rheumatic fever. You auscultate her heart and hear the following:

Discussion questions for the murmur of mitral stenosis:

1. Ask before playing audio: Based on the case what do you think the diagnosis may be?
2. What are you hearing regarding heart sounds? S1 is intensified. S2 is normal.
3. Describe the murmur. This is a diastolic murmur, best heard over the apex with an opening snap.
4. Based on these findings (a diastolic murmur heard over the apex) what is the diagnosis? Mitral stenosis
5. What position should the patient be sitting in to best hear the murmur of MS? Left lateral decubitus utilizing the bell of the stethoscope
6. Where is this murmur usually heard and where does it tend to radiate? Mitral stenosis tends not to radiate.

**CARDIAC EXAMINATION CHECKLIST**

|  | **Cardiac and Pulse Exam** |  |
| --- | --- | --- |
|  | **INSPECTION: Inspection**: Inspect skin for color, cyanosis, pallor. Observe patient’s breathing. Is it calm or labored? What is the temperature of the skin? Notice any skin changes such as xanthomata or rash. Inspect nails, inspect eyes, and mouth. Inspect the neck. Inspect chest configuration for pectus carinatum and excavatum |  |
| 1. P | **PALPATE arterial pulse:** Palpate the arterial pulse rate using radial artery. Determine the cardiac rhythm. Is it fast, slow, absent? Is it regular, regularly irregular, or irregularly irregular? Utilize your index and middle fingers, not your thumb |  |
|  | **Auscultate carotid arteries**. Instruct patient to hold breath while auscultating with the bell. Can be done with patient in sitting or supine position. |  |
|  | **Palpate the carotid pulses** (just medial to and below the angle of the jaw), one at a time, bilaterally. Can be done with patient in sitting or supine position. |  |
|  | **Palpate for the Point of Maximal Impulse-PMI (apical pulse) at the mitral (cardiac apex) area.** (5th intercostal space, mid-clavicular line) using only the pads of the fingers, not the fingertips. Patient should be lying in the supine position. If unable to palpate the PMI in the supine position, may need to ask the patient to “exhale and hold it” to locate the pulse, or roll to a left lateral decubitus position to locate the pulse. Once the apical impulse is detected, one can make finer measurements using fingertips and then 1 finger. |  |
|  | **Palpate the chest wall for thrills or heaves.** For heaves, press the ball of your hand on the chest to check for a buzzing or vibratory sensation from turbulence from a loud heart murmur. For thrills, place your palm and/or fingertips on each of the four cardiac areas. If present, you will feel a sustained impulse rhythmically lift your fingers. Presence of a thrill should prompt auscultation for a murmur in this area. |  |
|  | **Cardiac Auscultation: Utilize the 4 standard auscultation positions**  Auscultate each of the 4 areas of the heart using the diaphragm in the supine position  **1. Auscultate the aortic area:**  (2nd intercostal space at the right sternal border)  **2. Auscultate the pulmonic area:**  (2nd intercostal spaces at the left sternal border)  **3. Auscultate the tricuspid area:**  (4^th^ to 5^th^ intercostal space at left lower sternal border)  **4. Auscultate the mitral (cardiac apex) area:**  (5th intercostal space, mid-clavicular line, PMI) |  |
|  | **PALPATE arterial pulse:** Palpate the arterial pulse rate using radial artery. Determine the cardiac rhythm. Is it fast, slow, absent? Is it regular, regularly irregular, or irregularly irregular? Utilize your index and middle fingers, not your thumb |  |
|  | **Auscultate carotid arteries**. Instruct patient to hold breath while auscultating with the bell. Can be done with patient in sitting or supine position. |  |
|  | Auscultate each of the 4 areas of the heart using the diaphragm in the upright position  **1. Auscultate the aortic area:**  (2nd intercostal space at the right sternal border)  **2. Auscultate the pulmonic area:**  (2nd intercostal spaces at the left sternal border)  **3. Auscultate the tricuspid area:**  (4^th^ to 5^th^ intercostal space at left lower sternal border)  **4. Auscultate the mitral (cardiac apex) area:**  (5th intercostal space, mid-clavicular line, PMI) |  |
|  | **BLOOD VESSELS**  **Palpate Pulses**: Locate and palpate the following pulses bilaterally:   - Brachial - Radial - Popliteal - Dorsalis pedis - Posterior tibial |  |

**DEBRIEF**

- What was the most difficult part of this laboratory session?
- What do you feel like went particularly well, and what would you do to change the session in the future?
- What are some challenges that you encountered while auscultating heart sounds on the manikin? Did you feel comfortable differentiating between the heart sounds?
