# Supplementary material for: Cardiac Auscultation Lab Using a Heart Sounds Auscultation Simulation Manikin
Source: MedEdPORTAL. 2019 Oct 18;15:10839. doi: 10.15766/mep_2374-8265.10839 (PMC6974355; doi:10.15766/mep_2374-8265.10839)
Supplement: Supplementary file 1 — A. Heart Sounds - Programming List.docx B. Heart Sounds Lab - Facilitator Manual.docx C. Heart Sounds Lab - Student Manual.docx D. Post-Heart Sounds Lab Discussion.docx E. Session Feedback Form.docx [file mep-15-10839-s001.zip › C. Heart Sounds Lab - Student Manual.docx]

**Student Manual: Cardiac Exam and Heart Sounds Lab**

**Body Systems II: Cardiovascular, Respiratory, and Renal Systems**

**Session Title: CS Lab: Cardiac Exam and Heart Sounds**

**Description**

- Coaching and practicing the skill of performing the cardiac exam
- The session will begin with a lab and discussion on heart sounds.

**Readings**

Bates’ Guide to Physical Examination and History Taking, 11th edition:

pp 333-344 (Surface anatomy, cardiac chambers/valves, cardiac cycle, heart sounds, S3, S4, conduction system)

pp 360-366 (Techniques of Examination)

pp 367-368 (Carotid pulse, pulsus alternans, paradoxical pulse, thrills and bruits)

pp 369-385 (Heart Examination)

pp 500-501 (Peripheral pulses of arms)

pp 502-504 (Peripheral pulses of legs)

Heart Sounds:

<http://www.blaufuss.org> (optional)

Use this valuable web page to explore the different heart sounds and murmurs to understand the relevant anatomy and pathophysiology.

**Equipment**: Stethoscope

**Pre/Post-session assignments:**

No pre-session assignments

**Learning Objectives:**

By the end of this module, the learner will be able to:

1. Describe S1 and S2 with respect to intensity, location and splitting.
2. Identify and interpret physiological and pathological S1 and S2 including splitting in the context of a patient scenario and/or disease.
3. Identify S3 and S4 gallops and interpret S3 and S4 gallops in the context of a patient scenario and/or disease.
4. Describe location, timing and intensity of common systolic and diastolic heart murmurs.
5. Identify and interpret different systolic and diastolic murmurs in the context of a patient scenario and/or disease.
6. Demonstrate and distinguish auscultatory use of the diaphragm and bell of the stethoscope.

**PHYSICAL EXAM SESSION FORMAT:**

Your students will have 120 minutes to learn and practice the technique of performing a cardiac exam.

**0-10 minutes: Sign-in, brief orientation**

- Brief introduction of group members and facilitator
- Review of basic cardiac exam

**10-70 minutes: Heart Sounds Lab utilizing SAM II Cardiac Auscultation Manikin**

**70-110 minutes: Practice cases utilizing direct auscultation of SAM II simulator; Students practice the cardiac physical exam and get feedback on skill while waiting to use the manikin**

- Students should pair up and find an examination table on which to examine their partner.
- Students should practice their interpersonal skills by examining their partner in the role of a physician.
- Students should make certain to practice draping their partner.
- Students will utilize the auscultation manikin as a team of 4 (2 student pairs) to further analyze two additional cases.

**110-120 minutes: Debrief**

- Review the strengths and difficulties encountered by the students during the exam.
- Wrap up and final thoughts

# Cardiac Auscultation Algorithm

The SAM II Cardiac Auscultation Manikin is the basis for this heart sounds lab. Below are the steps to locating the audio for a variety of normal and abnormal heart sounds. Each section is introduced with a small case and is followed by discussion questions that should be answered while examining each of the heart sounds. In order to improve clinical reasoning, the students should predict which heart sounds are expected for each clinical scenario prior to listening to the sounds. Once the sound has been identified, a series of discussion questions follow.

Accentuated by Maneuver?

Rate?

(Fast/Normal/

Slow)

Rhythm?

(Regular, Irregular)

Murmur?

(Yes or No)

+ Murmur

Location?

Systolic/Diastolic?

Shape?

Extra Sounds?

(Systole/Diastole)

**Case #1**

Ms. Jennifer Alvarez, whom you had seen last year for her pre-exercise clearance, is back for her routine yearly examination. You listen to her heart and hear the following:

1. Based on the case what heart sounds do you expect to hear?
2. What sound are you hearing?

**Case #1**

Ms. Jennifer Alvarez, whom you had seen last year for her pre-exercise clearance, is back for her routine yearly examination. You listen to her heart and hear the following:

Discussion questions as you are examining the normal heart sounds:

1. How can you tell the first heart sound from the second?
2. Where is S1 best heard?
3. What does S1 signify? What is happening anatomically during S1?
4. What does S2 signify? What is happening anatomically during S2?
5. Where is the S2 best heard?
6. What do you hear now? How is it different?

**Case #2**

As your auscultation skills improve, you listen to Ms. Alvarez’s heart and hear the following:

1. What are you hearing?
2. How does this heart sound differ from the previous case?

**Case #2**

As your auscultation skills improve, you listen to Ms. Alvarez’s heart and hear the following:

Discussion questions as you are examining the physiologic split of S2:

1. What position do you have the patient lying in to appreciate this sound the best?
2. What part of the stethoscope would you use to hear this sound the best?
3. Where do you place the stethoscope to best hear this sound?
4. What causes a split S2?
5. Why does the S2 change with inspiration and expiration?

**Case #3**

A 67-year-old man with a history of a myocardial infarction 6 months ago now presents with pedal edema, orthopnea, decreasing exercise tolerance with dyspnea on walking 2 blocks and you hear the following heart sounds:

1. Based on the case what heart sounds do you expect to hear?
2. What sound are you hearing?

**Case #3**

A 67-year-old man with a history of a myocardial infarction 6 months ago now presents with pedal edema, orthopnea, decreasing exercise tolerance with dyspnea on walking 2 blocks and you hear the following heart sounds:

Discussion questions as you are examining the S3 gallop:

1. How does the presence of an S3 affect the sound of the S1 and S2?
2. What is an S3?
3. When in the cardiac cycle does an S3 occur?
4. Where on the chest wall is an S3 best heard?
5. What part of the stethoscope do you use to best hear an S3 and why?
6. How is the patient positioned to best hear an S3?
7. What does the presence of an S3 indicate clinically in a pediatric patient or athlete?
8. What does the presence of an S3 indicate clinically in this hypertensive patient?

**Case #4**

A 72-year-old woman with long-standing hypertension presents for her routine examination. Her blood pressure is still elevated at 160/94. You hear the following heart sounds:

1. Based on the case what heart sounds do you expect to hear?
2. What sound are you hearing?

**Case #4**

A 72-year-old woman with long-standing hypertension presents for her routine examination. Her blood pressure is still elevated at 160/94. You hear the following heart sounds:

Discussion questions as you are examining S4 gallop:

1. How does the presence of an S4 affect the sound of the S1 and S2?
2. What is an S4?
3. When in the cardiac cycle does and S4 occur?
4. Where on the chest wall is an S4 best heard?
5. What part of the stethoscope do you use to best hear an S4 and why?
6. How is the patient positioned to best hear an S4?
7. What does an S4 indicate clinically?

**Case #5**

A 12-year-old boy with sickle cell disease is admitted with an acute painful crisis. He is anemic and his hemoglobin is 5.2gm/dl. While listening to his heart you hear the following murmur:

1. Based on the case what heart sounds do you expect to hear?
2. What sound are you hearing?

**Case #5**

A 12-year-old boy with sickle cell disease is admitted with an acute painful crisis. He is anemic and his hemoglobin is 5.2gm/dl. While listening to his heart you hear the following:

Discussion questions for innocent murmur:

1. When does an innocent murmur occur during the cardiac cycle?
2. Where is an innocent murmur best heard?
3. What is the intensity or grading of functional murmurs? What does this mean?
4. What maneuvers decrease the intensity of a functional murmur?
5. What are some causes of functional murmurs?

**Case #6**

A 76-year-old woman with long-standing hypertension complains of substernal chest pain on walking 3 blocks that lasts for 5 minutes and then resolves when she stops walking. She also has dyspnea on exertion. While listening to his heart you hear the following:

1. Based on the case what heart sounds do you expect to hear?
2. What sound are you hearing?

**Case #6**

A 76-year-old woman with long-standing hypertension complains of substernal chest pain on walking 3 blocks that lasts for 5 minutes and then resolves when she stops walking. She also has dyspnea on exertion. While listening to his heart you hear the following:

Discussion questions for the murmur of aortic stenosis:

1. What are you hearing regarding heart sounds?
2. Describe the murmur.
3. Based on these findings what is the diagnosis?
4. What position should the patient be sitting in to best hear this murmur?
5. Where is this murmur usually heard and where does it tend to radiate?
6. What would you find on palpating her PMI?
7. What would you find on palpating her carotids?
8. The patient now returns several months later after passing out. How does this heart sound differ from the previous one?

**Case #7**

A 46yo male recently emigrated from North Africa to the United States and wants to establish primary care. His past medical history is significant for rheumatic fever as a child. You listen to his heart and hear the following:

1. Based on the case what heart sounds do you expect to hear?
2. What sound are you hearing?

**Case #7**

A 46yo male recently emigrated from North Africa to the United States and wants to establish primary care. His past medical history is significant for rheumatic fever as a child. You listen to his heart and hear the following:

Discussion questions for the murmur of aortic regurgitation:

1. Describe the murmur.
2. Based on these findings (a blowing decrescendo diastolic murmur) what is the diagnosis?
3. What position should the patient be sitting in to best hear the murmur of AR?
4. Where is this murmur usually heard and where does it tend to radiate?

**Case #8**

A 14-year-old boy is concerned because his 20-year-old cousin was recently told to stop playing College Varsity basketball. You listen to his heart and hear the following:

1. Based on the case what do you think the diagnosis may be?
2. What are you hearing regarding heart sounds?

**Case #8**

A 14-year-old boy is concerned because his 20-year-old cousin was recently told to stop playing College Varsity basketball. You listen to his heart and hear the following:

Discussion questions for the murmurs of hypertrophic cardiomyopathy:

1. What are you hearing regarding heart sounds?
2. Describe the murmur.
3. Based on these findings (a harsh shaped mid-systolic murmur in the aortic area with a diastolic murmur in the mitral area) what is the diagnosis?
4. Why are there 2 murmurs in this condition?
5. What position should the patient be sitting in to best hear the murmur of HOCM?
6. Where is this murmur usually heard and where does it tend to radiate?
7. What does handgrip, squatting and leg elevation do to this murmur? What about Valsalva strain? Why?

**SAM II auscultation manikin Case #1**

A 35yo female with a history of mitral valve prolapse since her teenage years. She presents with worsening dyspnea on exertion, fatigue, and decreased exercise tolerance. You auscultate her heart and hear the following:

1. Based on the case what do you think the diagnosis may be?
2. What are you hearing regarding heart sounds?

**SAM II auscultation manikin Case #1**

A 35yo female with a history of mitral valve prolapse since her teenage years. She presents with worsening dyspnea on exertion, fatigue, and decreased exercise tolerance. You auscultate her heart and hear the following:

Discussion questions for the murmur of mitral regurgitation:

1. What are you hearing regarding heart sounds?
2. Describe the murmur.
3. Based on these findings (a rectangular shaped holosystolic murmur) what is the diagnosis?
4. What position should the patient be sitting in to best hear the murmur of MR?
5. Where is this murmur usually heard and where does it tend to radiate?
6. What would bilateral increased handgrip do to this murmur? Why?
7. What do you hear now? How does the sound and radiation differ from the previous heart sound?

**SAM II auscultation manikin Case #2**

A 36yo female from Haiti presents to your office. She states she has a past medical history of rheumatic fever. You auscultate her heart and hear the following:

1. Based on the case what do you think the diagnosis may be?
2. What are you hearing regarding heart sounds?

**SAM II auscultation manikin Case #2**

A 36yo female from Haiti presents to your office. She states she has a past medical history of rheumatic fever. You auscultate her heart and hear the following:

Discussion questions for the murmur of mitral stenosis:

1. What are you hearing regarding heart sounds?
2. Describe the murmur.
3. Based on these findings (a diastolic murmur heard over the apex) what is the diagnosis?
4. What position should the patient be sitting in to best hear the murmur of MS?
5. Where is this murmur usually heard and where does it tend to radiate?
