# Supplementary material for: Cardiac Auscultation Lab Using a Heart Sounds Auscultation Simulation Manikin
Source: MedEdPORTAL. 2019 Oct 18;15:10839. doi: 10.15766/mep_2374-8265.10839 (PMC6974355; doi:10.15766/mep_2374-8265.10839)
Supplement: Supplementary file 1 — A. Heart Sounds - Programming List.docx B. Heart Sounds Lab - Facilitator Manual.docx C. Heart Sounds Lab - Student Manual.docx D. Post-Heart Sounds Lab Discussion.docx E. Session Feedback Form.docx [file mep-15-10839-s001.zip › E. Student Feedback Form.docx]

Heart Sounds Lab and Cardiac Exam (Clinical Skills Lab)

**Heart Sounds Lab and Cardiac Exam (Clinical Skills Lab)**
Please rate the effectiveness of this activity in facilitating your learning. Include in your consideration the assigned pre-readings, your level of engagement, and the clarity of the learning objectives. Please select a rating (radio button) below. A space for written comments is also provided. Written comments are valuable but are not required - the written comment box is optional.

- Extremely effective
- Very effective
- Average
- Somewhat effective
- Not at all effective

Comment on **Heart Sounds Lab and Cardiac Exam (Clinical Skills Lab)**

________________________________________________________________

________________________________________________________________

________________________________________________________________

________________________________________________________________

________________________________________________________________
